# PI3K/mTOR inhibition of IDH1 mutant glioma leads to reduced 2HG production that is associated with increased survival

**DOI:** 10.1038/s41598-019-47021-x

**Published:** 2019-07-19

**Authors:** Georgios Batsios, Pavithra Viswanath, Elavarasan Subramani, Chloe Najac, Anne Marie Gillespie, Romelyn Delos Santos, Abigail R. Molloy, Russell O. Pieper, Sabrina M. Ronen

**Affiliations:** 1 Department of Radiology and Biomedical Imaging, Mission Bay Campus, 1700 4th Street, Byers Hall, University of California, 94158 San Francisco, CA United States; 2 Department of Neurological Surgery, Helen Diller Research Center, 1450 3rd Street, University of California, 94143 San Francisco, CA United States; 3 Brain Tumor Research Center, Helen Diller Family Cancer Research Building, 1450 3rd Street, University of California, 94158 San Francisco, CA United States

**Keywords:** Cancer metabolism, Targeted therapies, Cancer imaging, CNS cancer

## Abstract

70–90% of low-grade gliomas and secondary glioblastomas are characterized by mutations in isocitrate dehydrogenase 1 (IDHmut). IDHmut produces the oncometabolite 2-hydroxyglutarate (2HG), which drives tumorigenesis in these tumors. The phosphoinositide-3-kinase (PI3K)/mammalian target of rapamycin (mTOR) pathway represents an attractive therapeutic target for IDHmut gliomas, but noninvasive indicators of drug target modulation are lacking. The goal of this study was therefore to identify magnetic resonance spectroscopy (MRS)-detectable metabolic biomarkers associated with IDHmut glioma response to the dual PI3K/(mTOR) inhibitor XL765. ^1^H-MRS of two cell lines genetically modified to express IDHmut showed that XL765 induced a significant reduction in several intracellular metabolites including 2HG. Importantly, examination of an orthotopic IDHmut tumor model showed that enhanced animal survival following XL765 treatment was associated with a significant *in vivo* ^1^H-MRS detectable reduction in 2HG but not with significant inhibition in tumor growth. Further validation is required, but our results indicate that 2HG could serve as a potential noninvasive MRS-detectable metabolic biomarker of IDHmut glioma response to PI3K/mTOR inhibition.

## Introduction

Gliomas are the most common type of brain tumor, representing 80% of all diagnosed malignant central nervous system tumors in the United States^1^. According to the World Health Organization, they are classified into low-grade gliomas (LGG; astrocytoma and oligodendroglioma) and the high-grade glioblastoma (GBM)^2,3^. GBM always presents as Grade IV and is the most aggressive form of glioma with an average survival of 15 months. Astrocytoma and oligodendroglioma tumors typically present as Grades II or III, and in the case of astrocytoma can also upgrade to Grade IV secondary GBM. They are characterized by a slower growth rate and a longer average survival of ~10 years^4–6^. Astrocytoma and oligodendroglioma tumors also affect patients at a younger age^1^. Traditionally this classification was based on histopathological analysis. However, recent understanding of the molecular genetic events that drive gliomagenesis^2,7^ led in 2016 to the WHO’s updated classification of gliomas^3^. In this context, the main characteristic of ~70–90% LGGs and secondary GBM is the presence of a mutation in the cytosolic form of isocitrate dehydrogenase 1 (IDHmut)^8^. In contrast, primary GBMs are now defined as wild-type for isocitrate dehydrogenase 1 (IDH1).

Wild type IDH1 catalyzes the oxidative decarboxylation of isocitrate to α-ketoglutarate (α-KG). Mutation occurs as a single amino acid substitution in the active site of IDH^9,10^, and the IDHmut enzyme catalyzes the reduction of α-KG to 2-hydroxyglutarate (2HG)^11,12^. 2HG has been shown to inhibit the activity of α-KG-dependent dioxygenases such as histone demethylases, prolyl hydroxylases and TET family of 5-methylcytosine hydroxylases^13^. This inhibition leads to alterations in cell signaling and gene expression that drive tumorigenesis^14,15^. As such, IDHmut is considered a ‘driver’ mutation in the development of LGGs, and 2HG is considered an oncometabolite. Interestingly, beyond 2HG production, IDHmut tumors also show a broader metabolic reprogramming that differs from that observed in primary GBM^9^.

In the clinical setting, non-invasive imaging is essential for the diagnosis and monitoring of brain tumors in patients. The most widely used imaging modality is magnetic resonance imaging (MRI)^16,17^. T2-weighted imaging, T1-weighted imaging post Gadolinium contrast agent injection, and fluid attenuated inversion recovery (FLAIR) imaging provide anatomical and structural information about the tumor and its surrounding area. In addition, perfusion-weighted imaging is used to assess tumor vascularity, and diffusion-weighted imaging is used to assess tumor cellularity^18,19^. Finally, MR spectroscopy (MRS) is used to provide information on tumor metabolism. ^1^H MRS is able to monitor steady state metabolite concentrations^20–23^. Complementary to ^1^H MRS, ^13^C MRS can be used to determine metabolic fluxes by assessing the fate of ^13^C-labeled metabolites, although this approach is most frequently used in the preclinical setting^24^. The metabolic information provided by ^1^H MRS has been shown to contribute to detection and characterization of brain tumors^25,26^. Most notably, elevated levels of choline-containing metabolites together with reduced levels of N-acetylasparate distinguish regions of tumor from normal brain. Lesions with elevated lactate and lipid have been shown to have higher-grade histology with shorter progression-free and overall survival, whereas elevated myo-inositol plus glycine relative to creatine is associated with low-grade astrocytomas^24^. Finally, detectable levels of 2HG are associated with IDHmut tumors for both oligodendrogliomas and astrocytomas^27^.

Current standard of care for high grade GBM is safe surgical resection, followed by radiotherapy and temozolomide (TMZ) treatment^28,29^. In contrast, in the case LGGs there is no standard of care. The current therapeutic approach for LGG includes maximal safe resection with subsequent treatment ranging from active surveillance by MRI to chemotherapy and radiation. According to Bush *et al*.^30^, if the patient is younger than 40 and has a gross total resection the patient is considered ‘low-risk’ and will undergo MRI surveillance only, while patients older than 40 or having a subtotal resection, are considered ‘high-risk’ and further treatment with radiation and chemotherapy should be considered. In addition to traditional chemotherapies (temozolomide, procarbazine, lomustine and vincristine)^6,31^ that affect all dividing cells, therapeutic strategies targeting biologically-relevant growth factors or cell signaling mediators that are specifically altered in cancer are also currently being considered^32–34^. One such cell signaling pathway is the phosphatidylinositol 3-kinase (PI3K)/mammalian target of rapamycin (mTOR) pathway.

The PI3K/mTOR pathway controls cell survival, proliferation and apoptosis^35–37^. Activation of the pathway is frequently detected in LGG and secondary glioblastoma, either as a result of enhanced platelet-derived growth factor (PDGF) signaling (via receptor amplification/mutation or ligand overexpression) or due to inactivation of the regulatory inhibitor PTEN (via PTEN promoter methylation and silencing)^38–40^. It has also been reported that LGG tumors from patients treated with TMZ who present with recurrent gliomas show activation of the PI3K/mTOR pathway, likely as a result of treatment^41,42^. Importantly, activation of the PI3K/mTOR pathway is correlated with worse LGG patient survival^43^. At the same time, inhibitors of the PI3K/mTOR pathway investigated in preclinical studies as well as in clinical trials in GBM and other tumor types, show improved median survival, reduced local and metastatic growth, and tumor growth inhibition^33,44–48^. Based on these earlier findings, inhibitors of the PI3K/mTOR pathway have entered clinical trials for LGG (NCT02023905, NCT01316809).

**Figure 1:**
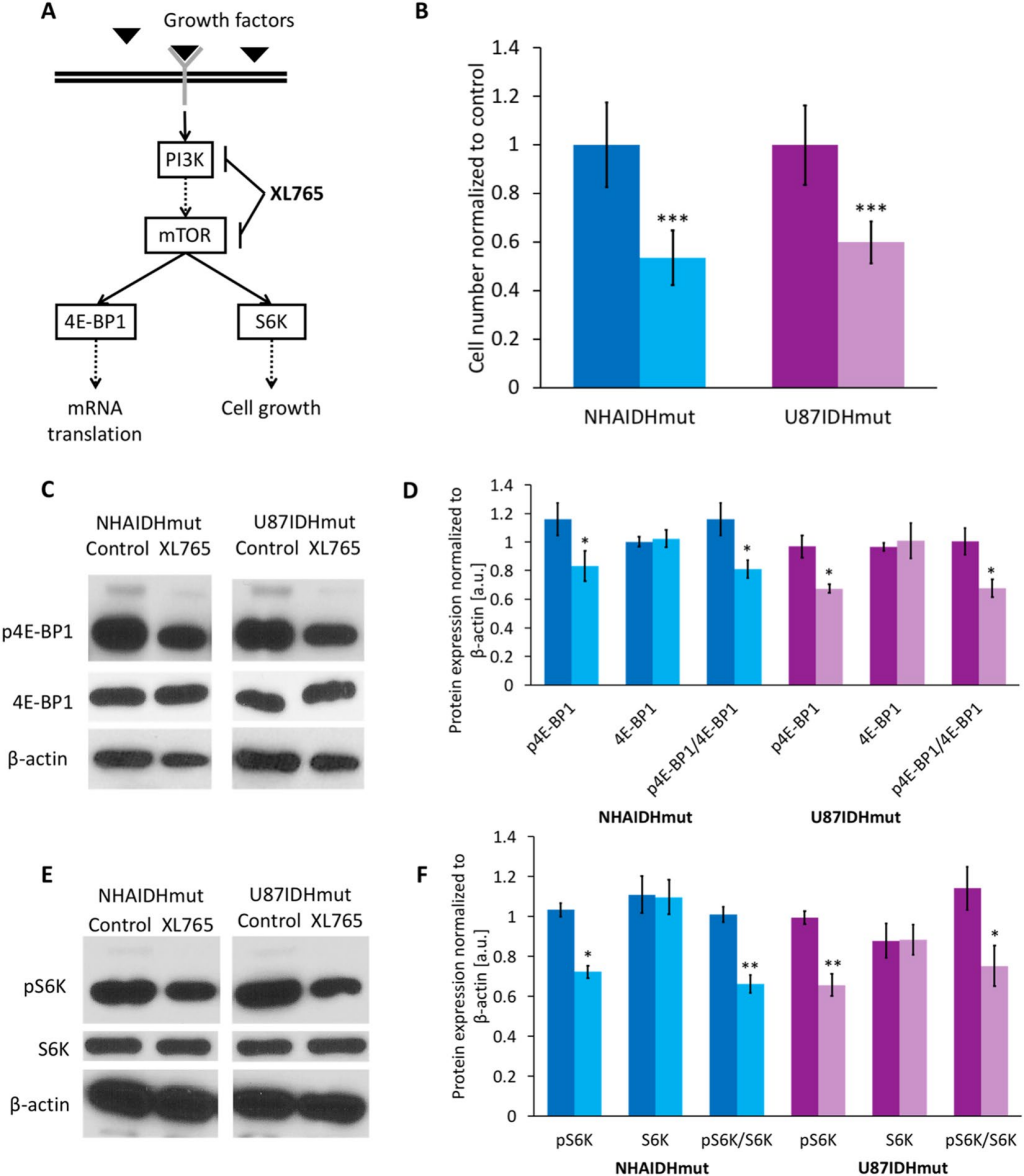
Validation of downregulation by XL765 of PI3K/mTOR pathway signaling by western blot. **(A)** Phosphoinositide-3-kinase (PI3K)/mammalian target of rapamycin (mTOR) signaling pathway. 4E-Binding protein 1 (4E-BP1) and S6 kinase (S6K) is a downstream target of mTOR. Solid lines: direct interaction; dotted lines: multistep interaction. **(B)** Quantification of cell number normalized to control for NHAIDHmut (Dark/light blue) and U87IDHmut (Dark/light purple). **(C)** Cropped western blots of phosphorylated 4E-BP1, 4E-BP1 and β-actin for NHAIDHmut (left) and U87IDHmut (right) model for control and XL765 treatment. Complete blots can be seen in Supplementary Fig. 1. **(D)** Quantification of p4E-BP1, 4E-BP1 and their ratio (p4E-BP1/4E-BP1) for NHAIDHmut and U87IDHmut model. **(E)** Cropped western blots of phosphorylated S6K, S6K and β-actin for NHAIDHmut (left) and U87IDHmut (right) model for control and XL765 treatment. Complete blots can be seen in Supplementary Fig. 2. **(F)** Quantification of pS6K, S6K and their ratio (pS6K/S6K) for NHAIDHmut and U87IDHmut model. Data are normalized to β-actin level. NHAIDHmut cell were treated with 32 μM XL765 for 72 h and U87IDHmut with 12 μM XL765 for 24 h. DMSO was used as vehicle control (NHAIDHmut: 0.16% for 72 h and U87IDHmut: 0.06% for 24 h). Dark blue/purple bar: Control; Light blue/purple bar: XL765-treated. *p < 0.05, **p < 0.01, ***p < 0.001.

However, PI3K inhibitors often induce tumor stasis rather than shrinkage^46–48^. Evaluation of tumor volume therefore is not sufficient to provide rapid and conclusive evidence of drug target engagement or response. The goal of our study was therefore to identify potential MRS-based metabolic biomarkers of LGG response to treatment with a PI3K/mTOR inhibitor. To this end, we investigated two genetically engineered cell models that express IDHmut^49^ and assessed the impact of XL765 (Voxtalisib), a pan-class I PI3K, mTORC1, and mTORC2 inhibitor (dual PI3K/mTOR inhibitor) (Fig. 1A), on their MRS-detectable metabolic profile^44,50^. Additionally, we verified our findings in an orthotopic mouse model *in vivo*. We found that in both cells and tumors XL765 treatment was associated with inhibition of cell proliferation and enhanced animal survival. This effect was associated with a reduction in ^1^H MRS-detectable steady state levels of 2HG, independent of tumor size. Our findings indicate that MRS could help assess the efficacy of PI3K inhibitors in IDHmut tumors and possibly predict treatment outcome.

## Results

### XL765 treatment inhibits 4E-BP1 and S6K phosphorylation downstream of PI3K/mTOR signaling in low-grade gliomas

We investigated immortalized normal human astrocytes (NHA) and U87 cells genetically engineered to express IDHmut (NHAIDHmut and U87IDHmut) as described previously^49^. First, we examined the effect of inhibiting PI3K/mTOR signaling using the dual PI3K/mTOR inhibitor XL765 (see Fig. 1A) on cell proliferation. Each cell line was treated with a dose that resulted in approximately 50% reduction in cell number. NHAIDHmut cells were treated with 32 μM XL765 for 72 h leading to 55.7 ± 16% inhibition while U87IDHmut cells were treated with 12 μM XL765 for 24 h, resulting in 61.0 ± 10.6% inhibition (Fig. 1B). We confirmed inhibition of the PI3K/mTOR pathway following XL765 treatment by examining the phosphorylation status of 4E-BP1 (p4E-BP1) and S6K (pS6K) downstream of PI3K/mTOR signaling (see Fig. 1A). XL765 treatment induced a significant reduction in p4E-BP1 and pS6K levels in both NHAIDHmut and U87IDHmut cells (Fig. 1C–F and Supplementary Figs 1 and 2). Importantly, XL765 treatment did not alter total levels of 4E-BP1 and S6K. Taken together, these results confirmed that XL765 inhibited PI3K/mTOR signaling in our low-grade glioma models.

### XL765 treatment alters ^1^H MRS-detectable metabolites levels in low-grade gliomas

**Figure 2:**
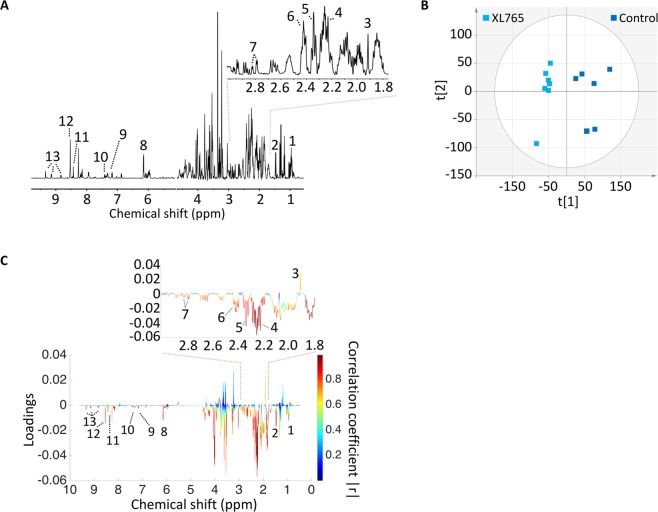
Multivariate analysis shows a discrimination between XL765-treated and control group in NHAIDHmut cells. **(A)** A typical 500 MHz ^1^H NMR spectrum of the water fraction of control cell extract. Numbers indicate the following metabolites: 1. Leucine, 2. Alanine, 3. Acetate, 4. 2HG, 5. Glutamate, 6. Glutamine, 7. Aspartate, 8. AXP, 9. Tyrosine, 10. Phenylalanine, 11. Formate, 12. NAD+/NADP+ or NADH/NADPH, 13. NAD+/NADP+. **(B)** Two-dimensional scatter plot of PCA shows discrimination of control (dark blue) and XL765-treated (light blue) groups. **(C)** Loading plot for ^1^H NMR. The correlation coefficient corresponding to OPLS-DA model is represented as color map. Numbers indicate the same metabolites as in **(A)** and had |r| > 0.8. OPLS-DA: Orthogonal partial least squares discriminant analysis.

**Figure 3:**
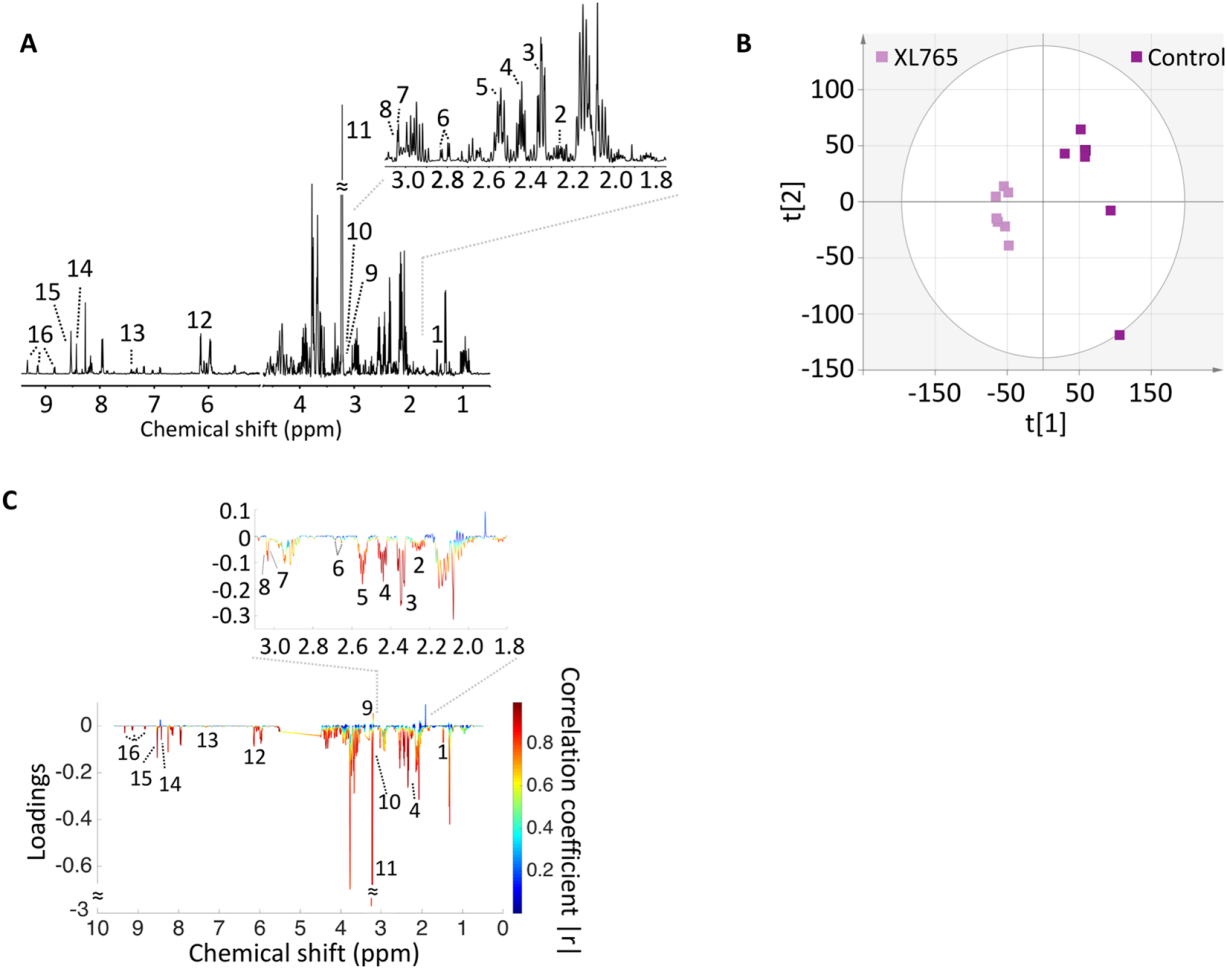
Multivariate analysis shows a discrimination between XL765-treated and control group in U87IDHmut cells. **(A)** A typical 500 MHz ^1^H NMR spectrum of the water fraction of control cell extract. Numbers indicate the following metabolites: 1. Alanine, 2. 2HG, 3. Glutamate, 4. Glutamine, 5. Glutathione, 6. Aspartate, 7. Creatine, 8. Phosphocreatine, 9. Choline, 10. Phosphocholine, 11. Glycerylphosphorylcholine, 12. AXP, 13. Phenylalanine, 14. Formate, 15. NAD+/NADP+ or NADH/NADPH, 16. NAD+/NADP+. **(B)** Two-dimensional scatter plot of PCA shows discrimination of control (dark purple) and XL765-treated (light purple) groups. **(C)** Loading plot for ^1^H NMR. The correlation coefficient corresponding to OPLS-DA model is represented as color map. Numbers indicate the same metabolites as in **(A)** and had |r| > 0.8. OPLS-DA: Orthogonal partial least squares discriminant analysis.

We then examined the effect of XL765 on steady-state metabolite levels using ^1^H MRS in the NHAIDHmut (Fig. 2A) and U87IDHmut (Fig. 3A) models. Multivariate analysis was used to compare the XL765-treated group and the control group. First, principle component analysis (PCA) was applied for an unbiased reduction of the complexity of the data. In both cell models PCA was able to reliably distinguish the XL765-treated group from the control group using only the first two principle components t[1] and t[2] (see Fig. 2B: NHAIDHmut, R2X (fraction of variation of X variables explained by model) = 0.407, Q2 (goodness of prediction) = 0.0484; N = 6 for control and N = 7 for XL765; Fig. 3B: U87IDHmut, R2X = 0.515, Q2 = −0.0102, N = 6 for control and N = 7 for XL765). Next, the data was analyzed using a supervised orthogonal partial least squares discriminant analysis (OPLS-DA) (Supplementary Fig. 3; NHAIDHmut R2Y (fraction of variation of Y variables explained by model) = 0.989, Q2 = 0.872; and U87IDHmut, R2Y = 0.934, Q2 = 0.901) and S-plots derived from this classification (NHAIDHmut: Fig. 2C; U87IDHmut: Fig. 3C). The correlation coefficient (|r|) of the metabolites responsible for the discrimination between the two groups is illustrated by the color of the points on the S-plot while the relevance to the model (loadings) is indicated by the signal amplitude. Based on this information, we focused on metabolites with |r| higher than 0.8. The cellular concentrations of these metabolites were then subjected to univariate analysis and our findings are summarized in Table 1 (NHAIDHmut) and 2 (U87IDHmut). As shown in the S-plots in Figs 2C and 3C as well as Tables 1 and 2, several metabolites were commonly altered in both cell lines. Importantly, levels of alanine, 2HG, glutamate, glutamine and aspartate were significantly reduced in XL765-treated cells in both of our models, thus identifying potentially translational metabolic biomarkers of IDHmut glioma cell response to PI3K/mTOR inhibition.

**Table 1:** Quantitative comparison of metabolites quantified from the aqueous phase of control and XL765-treated NHAIDHmut cell extract that presented correlation coefficient |r| higher than 0.8 in the S-plot (Fig. 2C).

Metabolite	Control group concentration (mean ± STD) [fmol/cell]	XL765 group concentration (mean ± STD) [fmol/cell]	p-value	Change under XL765 treatment [%]
Leucine	0.29 ± 0.07	0.15 ± 0.03	<0.001	−49.10
**Alanine**	0.42 ± 0.16	0.11 ± 0.02	0.002	−72.57
Acetate	0.34 ± 0.14	0.71 ± 0.27	0.004	113.14
**2HG**	4.71 ± 1.15	0.42 ± 0.27	<0.001	−91.06
**Glutamate**	2.32 ± 0.13	0.75 ± 0.11	<0.001	−67.70
**Glutamine**	2.63 ± 0.75	1.31 ± 0.15	0.002	−50.17
**Aspartate**	1.10 ± 0.38	0.47 ± 0.05	0.003	−57.30
**AXP**	1.32 ± 0.23	0.70 ± 0.17	<0.001	−47.15
Tyrosine	0.38 ± 0.10	0.21 ± 0.03	0.002	−44.60
**Phenylalanine**	0.40 ± 0.09	0.15 ± 0.04	<0.001	−62.61
**Formate**	0.28 ± 0.06	0.11 ± 0.04	<0.001	−60.82
**NAD(P)H/+**	0.16 ± 0.03	0.08 ± 0.01	<0.001	−51.93
**NAD(P)+**	0.06 ± 0.01	0.03 ± 0.01	<0.001	−59.17

Metabolites in bold mark metabolites that are common in the two cell lines.

**Table 2:** Quantitative comparison of metabolites quantified from the aqueous phase of control and XL765-treated U87IDHmut cell extract that presented correlation coefficient |r| higher than 0.8 in the S-plot (Fig. 3C).

Metabolite	Control group concentration (mean ± STD) [fmol/cell]	XL765 group concentration (mean ± STD) [fmol/cell]	p-value	Change under XL765 treatment [%]
**Alanine**	0.82 ± 0.34	0.06 ± 0.04	<0.001	−92.13
**2HG**	1.49 ± 0.30	0.31 ± 0.09	<0.001	−79.38
**Glutamate**	4.47 ± 0.90	1.79 ± 0.40	<0.001	−60.04
**Glutamine**	5.26 ± 2.40	1.02 ± 0.63	<0.001	−80.56
Glutathione	0.67 ± 0.22	0.48 ± 0.10	0.025	−29.02
**Aspartate**	13.93 ± 5.15	3.30 ± 0.48	<0.001	−76.3
Creatine	0.67 ± 0.32	0.16 ± 0.06	0.001	−75.63
Phosphocreatine	0.41 ± 0.24	0.16 ± 0.06	0.060	−60.58
Choline	0.03 ± 0.04	0.16 ± 0.11	0.006	600.78
Phosphocholine	2.20 ± 1.49	0.18 ± 0.13	0.008	−70.99
Glycerylphosphorylcholine	8.34 ± 2.27	0.64 ± 0.24	<0.001	−48.45
**AXP**	4.44 ± 1.21	4.30 ± 1.22	<0.001	−57.02
**Phenylalanine**	1.04 ± 0.38	0.64 ± 0.21	0.010	−38.29
**Formate**	1.09 ± 0.26	0.51 ± 0.17	<0.001	−52.91
**NAD(P)H/+**	0.52 ± 0.16	0.19 ± 0.03	<0.001	−64.77
**NAD(P)+**	0.24 ± 0.11	0.08 ± 0.03	0.001	−64.66

Metabolites in bold mark metabolites that are common in the two cell lines.

### XL765 inhibits PI3K/mTOR signaling and prolongs animal survival in orthotopic tumor xenografts *in vivo*

**Figure 4:**
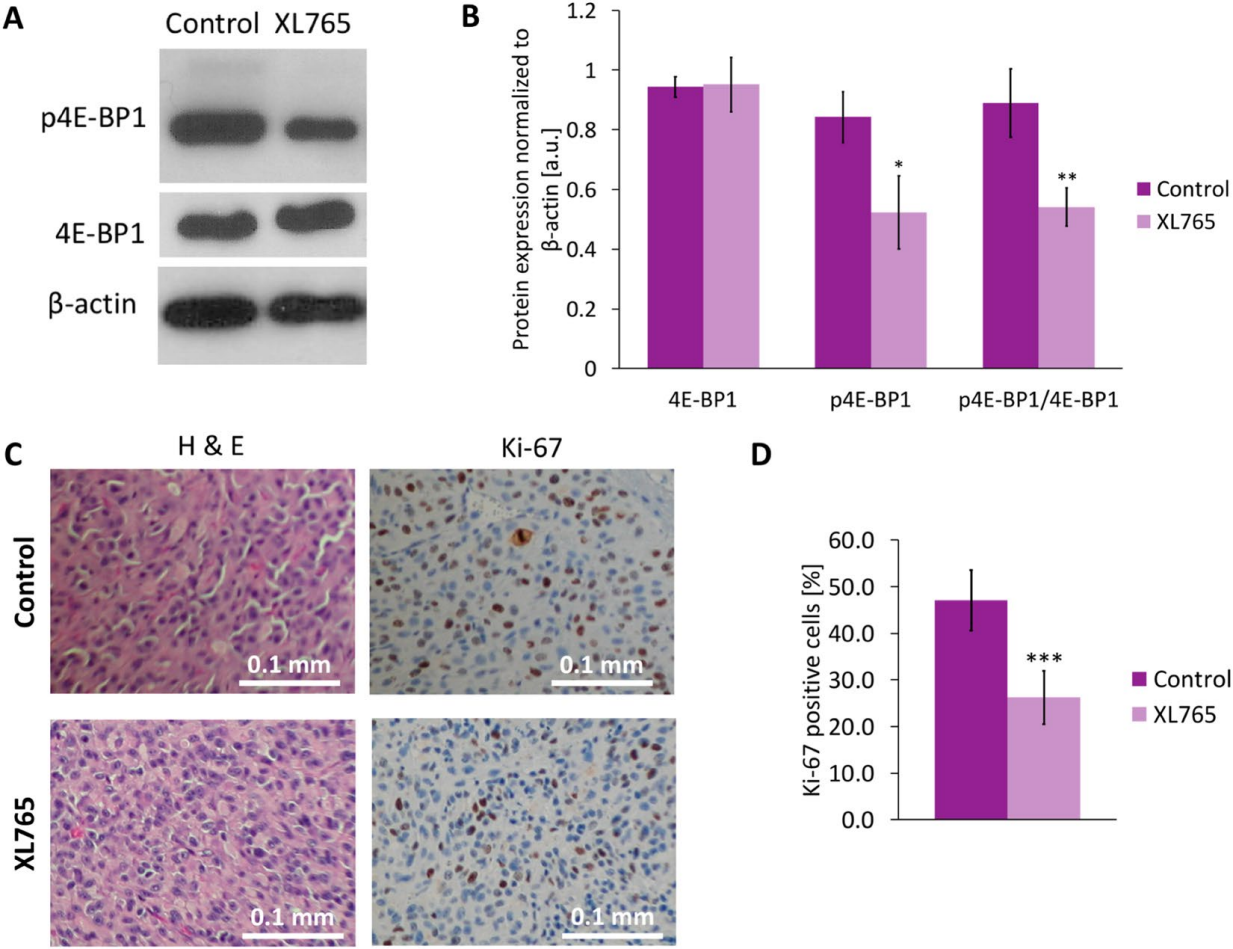
Validation in tissue samples of downregulation of PI3K/mTOR pathway signaling and reduction in cell proliferation under XL765 treatment. **(A)** Cropped western blots of phosphorylated 4E-BP1, 4E-BP1 and β-actin for tissues from control and XL765-treated mice. Complete blots can be seen in Supplementary Fig. 4. **(B)** Quantification of 4E-BP1, p4E-BP1 and their ratio (p4E-BP1/4E-BP1). Data are normalized to β-actin level. **(C)** Immunohistochemical analysis of control and XL765‐treated U87IDHmut tumors. Expression of Ki-67 for each treatment group at the end of the study. All histological images are the same magnification (×20). **(D)** Quantification Ki-67 positive cells normalized to total number of cells. Dark purple bar: Control; Light purple bar: XL765-treated. *p < 0.05, **p < 0.01, ***p < 0.001.

**Figure 5:**
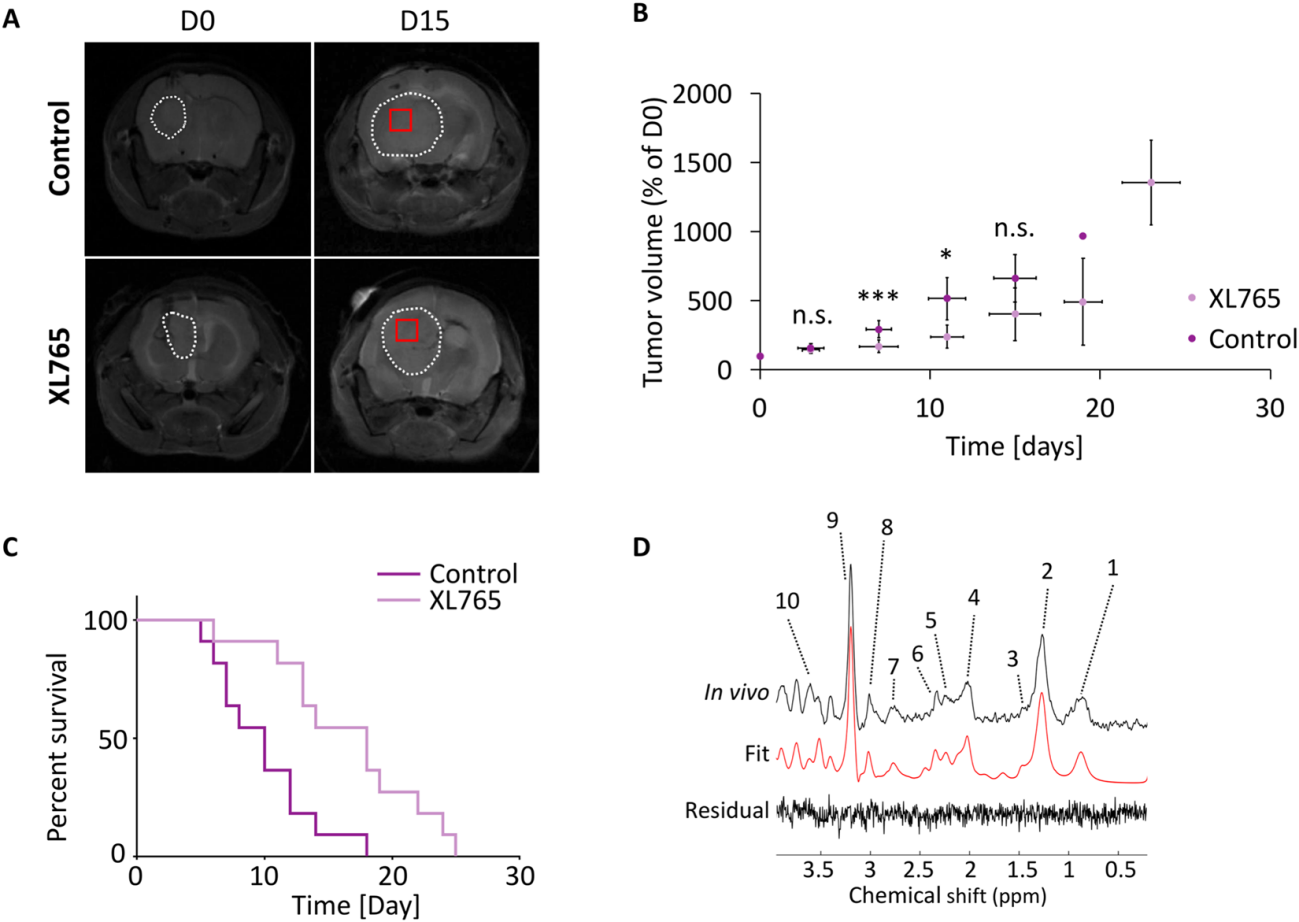
XL765 treatment increases animal survival without apparent tumor volume difference. **(A)** Axial T2-weighted images at D0 (first column) and D15 of treatment (second column) for control (top) and XL765-treated (bottom) U87IDHmut-tumor bearing mice brains. Tumor regions are contoured with a white line and the square box at D15 depicts the ^1^H-MRS voxel. Day 15 was the final day for both presented mice. Mice were treated with 30 mg/kg XL765 twice daily p.o. or with 10 mM HCl (control group). **(B)** Temporal evolution of average tumor volume normalized to D0. (*p < 0.05, ***p < 0.001, n.s.: not significant) **(C)** Kaplan–Meier survival curves comparing survival between XL765-treated animals (light gray line) and control (dark purple line) ones. **(D)** Representative spectra of a control animal, its LCModel fit and residual of the fit. Apodization of 5 Hz has been applied. Numbers indicate the following metabolites: 1. Lipids, 2. Lactate and Lipids, 3. Alanine, 4. N-acetylaspartate, 5. 2HG, 6. Glutamate and Glutamine, 7. Aspartate, 8. Total creatine, 9. Total choline and 10. Myo-Inositol.

Next, we assessed the impact of XL765 *in vivo* on U87IDHmut orthotopic tumor xenografts. Consistent with our findings in our cell models, XL765 reduced levels of p4E-BP1 without affecting total 4E-BP1 levels (Fig. 4A,B and Supplementary Fig. 4). Immunohistochemical analysis pointed to a significant reduction in Ki-67 staining in XL765-treated tumors (Fig. 4C,D). With regard to tumor growth, examination of T2-weighted MR images from control and XL765-treated animals (Fig. 5A) indicated that, although there was a significant reduction in tumor volume at day 8 and day 12, there was no significant difference at the majority of time-points (Fig. 5B). Importantly, XL765 treatment significantly prolonged animal survival as illustrated in the Kaplan-Meier survival plot (Fig. 5C; N = 11; χ = 8.555 and p = 0.003). Taken together, these results indicated that XL765 inhibited PI3K/mTOR signaling *in vivo* and pointed to a cytostatic effect of XL765 on tumor proliferation, consistent with previous studies indicating that PI3K/mTOR inhibition leads to tumor stasis^48^.

### XL765 treatment reduces ^1^H MRS-detectable 2HG levels *in vivo*

To assess whether XL765 treatment *in vivo* induced metabolic alterations similar to those observed in our cell studies, we acquired *in vivo* ^1^H spectra from control and XL765-treated tumors. Figure 5D shows a representative ^1^H spectrum from a control animal along with the spectral fit and residual signal from LCModel. We then quantified metabolites for which Cramer–Rao lower bounds (CRLB) were <40% (Table 3). Consistent with our cell studies, there was a significant drop in 2HG levels from 0.132 ± 0.010 a.u. in controls to 0.103 ± 0.008 a.u. in XL765-treated animal (N = 5 for control group, N = 6 for XL765-treated group, CRLB < 40%), as well as a significant drop in glutamate (from 0.127 ± 0.006 a.u. in controls to 0.101 ± 0.004 a.u. in XL765-treated animal; N = 9 for control group, N = 10 for XL765-treated group, CRLB < 25%). However, in contrast to our cell findings, there was a significant increase in glutamine levels while there was no change in alanine. Aspartate could not be quantified with any reasonable accuracy (CRLB > 40%) in more than 3 animals in XL765-treated tumors.

**Table 3:** Quantitative comparison of metabolites quantified using LCModel in *in vivo* collected ^1^H spectra from control and XL765-treated mice bearing intracranial U87IDHmut tumors.

Metabolite	CRLB [%]	Number of animals		Control group concentration normalized to total signal (mean ± STD) [a.u.]	XL765 group concentration normalized to total signal (mean ± STD) [a.u.]	p-value	Change under XL765 treatment [%]
		Control	XL765				
Alanine	30	9	7	0.063 ± 0.004	0.062 ± 0.003	0.53	−1.62
2HG	40	5	6	0.132 ± 0.010	0.103 ± 0.008	<0.001	−21.67
Glutamate	25	9	10	0.127 ± 0.006	0.101 ± 0.004	<0.001	−20.46
Glutamine	40	4	4	0.058 ± 0.008	0.088 ± 0.005	<0.001	52.58
Aspartate	40	4	2	0.073 ± 0.008	0.082 ± 0.016	—	—

**Figure 6:**
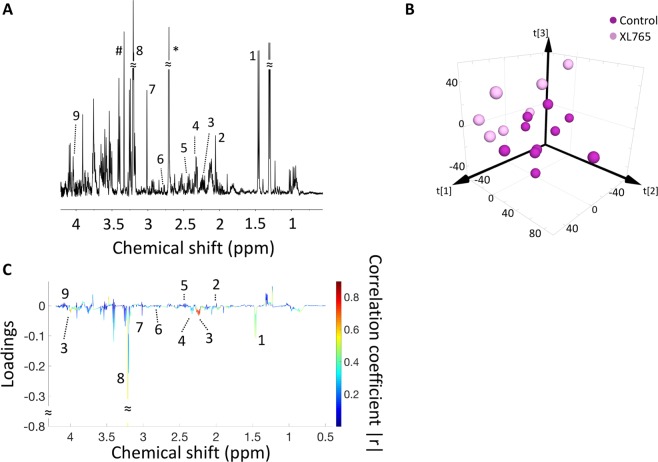
2HG discriminates control from XL765-treated U87IDHmut tumor extracts. **(A)** A typical 500 MHz ^1^H NMR spectrum of the water fraction of tumor extract from a control animal. Numbers indicate the following metabolites: 1. Alanine, 2. N-acetylaspartate, 3. 2HG, 4. Glutamate, 5. Glutamine, 6. Aspartate, 7. Total creatine, 8. Total choline, 9. Myo-Inositol. *: speak (2.7 ppm) related to protease inhibitor solution, ^#^: methanol residual. **(B)** Three-dimensional scatter plot of PCA shows discrimination of control (dark purple) and XL765-treated (light purple) groups. **(C)** Loading plot for ^1^H NMR. The correlation coefficient corresponding to OPLS-DA model is represented as color map. Numbers indicate the same metabolites as in **(A)**. OPLS-DA: Orthogonal partial least squares discriminant analysis.

Since the CRLBs used in the analysis of our *in vivo* MRS data were relatively high, and because there was some discrepancy between our cell and tumor data, we performed higher resolution *ex vivo* tumor extract studies to confirm our *in vivo* observations. ^1^H MRS data was recorded from control and XL765-treated tumor tissue extracts (Fig. 6A), and similar to our cell studies, unbiased multivariate analysis was then used to compare the XL765-treated group and the control group. PCA was able to reliably distinguish the XL765-treated group from the control group using the first three principle components t[1], t[2] and t[3] (Fig. 6B: R2X = 0.395, Q2 = 0.0208; N = 10 for control and 7 for XL765). Next, the data was analyzed using a supervised OPLS-DA analysis (Supplementary Fig. 3; R2Y = 0.929, Q2 = 0.241) and an S-plot derived from this classification (Fig. 6C). The only region with a correlation coefficient (|r|) higher than 0.8 was the 2.2–2.3 ppm which corresponds to 2HG. Univariate analysis of the 2HG concentration in the two groups confirmed a significant drop (p = 0.003) from 0.089 ± 0.045 a.u. to 0.027 ± 0.017 a.u. following XL765 treatment. Univariate analysis of the glutamate concentration in the two groups agreed with the multivariate S-plot analysis as there was no significant change (p = 0.32; 0.152 ± 0.032 a.u. and 0.187 ± 0.076 a.u. for control and XL765-treated group respectively). Taken together with the *in vivo* ^1^H MRS data, these results point to 2HG as a robust imaging biomarker of IDHmut glioma response to XL765 treatment.

### Production of 2HG from glucose and glutamine is reduced in treated cells

**Figure 7:**
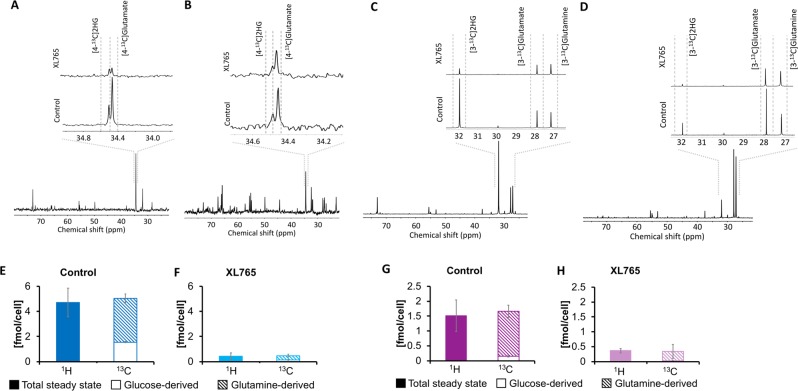
Glucose and glutamine flux to 2HG is reduced in NHAIDHmut and U87IDHmut cells under XL765 treatment. **(A**,**B)** Representative ^13^C-spectra ([1-^13^C]glucose labeling) of NHAIDHmut and U87IDHmut control cells respectively. Inserts: Expansion of [4-^13^C]2HG and [4-^13^C]glutamate region for XL765-treated (top) and control (bottom). **(C**,**D)** Representative ^13^C-spectra ([3-^13^C]glutamine labeling) of NHAIDHmut and U87IDHmut control cells respectively. Inserts: Expansion of [3-^13^C]2HG and [3-^13^C]glutamate region for XL765 treatment (top) and control (bottom). **(E–H)** Average 2HG produced from [1-^13^C]glucose and [3-^13^C]glutamine (^13^C) and total 2HG levels (^1^H) for control and XL765-treated NHAIDHmut cells **(E**,**F)** and U87IDHmut cells **(G**,**H)**. Dark blue/purple bar: Control; Light blue/purple bar: XL765-treated; Full bar: Steady state; Empty bar: Glucose derived metabolite; Hashed bar: Glutamine derived metabolite.

Previous work^24^ has shown that glucose and glutamine are the precursors of 2HG in IDHmut glioma cells. To further confirm our ^1^H MRS findings and assess alterations in metabolic fluxes from glucose and glutamine induced by XL765 treatment, we used ^13^C MRS to probe the fate of [1-^13^C]glucose and [3-^13^C]glutamine. Consistent with previous work^24^, our extracts demonstrated that [1-^13^C]glucose was incorporated into [4-^13^C]2HG (34.5 ppm) in both NHAIDHmut (Fig. 7A) and U87IDHmut (Fig. 7B) models while [3-^13^C]glutamine was incorporated into [3-^13^C]2HG (32 ppm, Fig. 7C,D). Our results indicate that, following XL765 treatment, flux from both glucose and glutamine towards 2HG was significantly reduced in both NHAIDHmut and U87IDHmut models, thus explaining the drop in 2HG steady state levels observed by ^1^H MRS. Specifically, in the NHAIDHmut model, 2HG produced from [1-^13^C]glucose dropped from 1.56 ± 0.05 fmol/cell in control cells to 0.12 ± 0.04 fmol/cell in XL765-treated cells (p < 0.001). We obtained similar results with [2-^13^C]glucose: 2HG levels dropped from 1.46 ± 0.26 fmol/cell in control to 0.13 ± 0.03 fmol/cell following XL765 treatment (p = 0.04; Supplementary Fig. 5; N = 3). [3-^13^C]glutamine-derived 2HG dropped from 3.50 ± 0.35 fmol/cell in NHAIDHmut control to 0.34 ± 0.12 fmol/cell in NHAIDHmut XL765-treated cells (p = 0.003; Fig. 7E,F, N = 3). Similarly, in U87IDHmut cells, 2HG produced from [1-^13^C]glucose dropped following XL765 treatment from 0.16 ± 0.04 fmol/cell to 0.03 ± 0.01 fmol/cell (p = 0.007), while flux from [3-^13^C]glutamine to 2HG dropped from 1.50 ± 0.20 fmol/cell to 0.32 ± 0.23 fmol/cell (p = 0.006) following XL765 treatment (Fig. 7G,H; N = 4 for [1-^13^C]glucose and N = 3 for [3-^13^C]glutamine). Similar results were obtained when flux from [2-^13^C]glucose to 2HG was calculated (0.23 ± 0.04 fmol/cell in U87IDHmut control dropped to 0.06 ± 0.04 fmol/cell in U87IDHmut XL765-treated cells; p = 0001; Supplementary Fig. 5, N = 4). Comparison of the steady-state concentration of 2HG determined from the ^1^H spectra to the combined ^13^C MRS values showed that the data were within experimental error, further validating our findings. Specifically, for NHAIDHmut the ^1^H MRS-derived steady state 2HG was 4.71 ± 1.15 fmol/cell for control and dropped to 0.42 ± 0.27 fmol/cell following XL765 treatment, while the combined ^13^C values were 5.05 ± 0.36 fmol/cell and dropped to 0.46 ± 0.12 fmol/cell (Fig. 7E,F). For U87IDHmut steady-state 2HG was 1.49 ± 0.30 fmol/cell in control and 0.31 ± 0.09 fmol/cell in XL765-treated, while the combined ^13^C values were 1.66 ± 0.20 fmol/cell and 0.34 ± 0.23 fmol/cell respectively (Fig. 7G,H).

## Discussion

Treatment of lower grade gliomas increasingly involves chemotherapy and radiotherapy^51–53^. In an effort to further improve outcomes for LGG patients, other therapeutic options are being considered. One such approach is to inhibit the PI3K/mTOR pathway^33^. PI3K/mTOR pathway activation has been linked with worse patient survival in LGGs^43^, supporting clinical trials of inhibitors of the pathway as a treatment regimen for those tumors (NCT02023905, NCT01316809). The outcomes of these trials remain to be determined, but studies in other tumor types have shown that PI3K/mTOR signaling inhibitors often lead to tumor stasis rather than shrinkage^46–48^. The goal of our study was therefore to identify potential metabolic imaging biomarkers that could help assess drug target engagement and the likely response of LGG to inhibitors of the PI3K/mTOR pathway.

To perform our studies, we chose to focus on monitoring the effect of a dual PI3K and mTOR inhibitor. This choice was driven by research in other tumor types that has shown that inhibition of mTOR only results in a feedback loop, activation of compensatory pathways, and tumor resistance^54–57^. More effective inhibition of tumor growth was achieved by simultaneously targeting both PI3K and mTOR. Additionally, it has been shown that dual PI3K/mTOR inhibitors, such as XL765, were more effective that individual PI3K or mTOR inhibitors combined^58^. Collectively such studies have led to the use of dual inhibitors in clinical phase I and II trials in a range of tumor types including GBM and, importantly, several such studies have resulted in clinically-relevant disease stability^50,59–64^ (NCT03522298, NCT01547546).

We assessed the effect of treatment in two genetically engineered cell models. These models grow reliably and reproducibly as cells and, in the case of the U87IDHmut model, also as tumors, providing us with a robust platform to assess the impact of our inhibitor. Our studies show that whereas response to XL765 in our IDHmut models is associated with a drop in several metabolites, only a drop in 2HG was also reliably detected in the *in vivo* setting. Future studies will focus on confirming our findings in patient-derived models, but it should be noted that few such models are available and they are typically challenging to grow consistently both as cells and *in vivo*. Nonetheless, the drop in 2HG was observed in both of our models, independent of their genetic background (one is a GBM and the other is a NHA) lending support to the reliability of our findings. Furthermore, our finding that 2HG is reduced following PI3K/mTOR inhibition is in agreement with the report from Hujber *et al*.^65^ who showed reduce 2HG production in IDHmut fibrosarcoma cells following mTOR inhibition.

Our observation that 2HG drops *in vivo* was independent of tumor size, but was associated with enhanced animal survival, pointing to the value of this biomarker as an indicator of PI3K inhibition and likely response to treatment. The drop in 2HG would serve as a biomarker that is specific to IDHmut LGG tumors. Studies of GBM tumors, including U87 tumors, previously performed by us and others detected changes in lactate production and phosphocholine levels, but changes in 2HG would not be expected in GBM as they do not harbor the IDH1 mutation and do not produce 2HG^66–68^.

The use of the genetically-engineered U87IDHmut model for *in vivo* validation of our findings could be considered a potential limitation of our study. However, it should be noted that there is a only a small number of patient-derived IDHmut glioma models that form orthotopic tumors *in vivo* (e.g MGG119 and MGG152 and BT142)^38,69^. In our prior studies, we have investigated the patient-derived BT142 model^24,69,70^. However, this model has lost 2HG production due to loss of the wild-type IDH allele, a phenomenon that has been shown to occur in IDH mutant gliomas^71^. In contrast, the U87IDHmut model is an easy to grow and very stable model that has been frequently used, not only by our group, but also in several other studies in the literature^72–76^. Importantly, we have previously demonstrated that 2HG levels in our U87IDHmut tumor tissue extracts (9.8 ± 1.6 μmol/g of tissue) were comparable to 2HG levels reported in patients (5–35 μmol/g)^77^. Furthermore, it should be noted that our metabolic findings in the U87IDHmut model differ significantly from that of a prior study which examined the response of IDH wild-type glioblastomas to XL765 treatment^67^. Based on these observations, we believe that the use of the U87IDHmut glioma model *in vivo* is justified for the purposes of the current study.

The mechanism by which inhibition of PI3K/mTOR signaling leads to a drop in 2HG remains to be fully elucidated. However, our ^13^C MRS studies point to a drop in both the flux of glucose and the flux of glutamine toward that metabolite. These findings are consistent with previous publications that have shown that the PI3K/mTOR pathway controls both glucose and glutamine uptake and metabolism^78–81^. Inhibition of PI3K/mTOR would thus inhibit glucose and glutamine metabolism providing a possible explanation for our observations.

Importantly, when considering translation of our findings, several approaches have now been optimized for 2HG detection at clinical field strengths as recently review by Leather *et al*.^27^. Each has its strengths and weaknesses^82–87^. A study using a standard single-voxel double echo point-resolved spectroscopy (PRESS) sequence with a short echo time (TE = 30 ms) at 3 T combined with a standard fitting algorithm (LCModel)^88^ can be readily implemented to detect 2HG but was associated with a 26% false positive detection rate in IDH wild type tumors due to the overlap of 2HG with glutamate and glutamine at 2.25 ppm^85^. An optimized PRESS sequence utilizing a longer TE (TE = 97 ms at 3 T or 78 ms at 7 T) can improve spectral resolution and reduce spectral overlap^82,89^ leading to 100% specificity and sensitivity for 2HG detection, but the method can lead to quantification errors due to the longer TE^90^. An alternative approach is the use of a 2D correlation spectroscopy (COSY) sequence at 3T^83,86^. The method counteracts the known confounding spectral overlap observed in 1D MRS spectra and allows for better separation of metabolites, but the clinical applicability of such a technique is limited due to the extended acquisition time, an important factor for incorporation into clinical practice. An alternative approach is the use of three dimensional MRS imaging approach combined with 2HG spectral editing, an approach which has been already applied for treatment response assessment in patients^34^. Finally, a study at 7 T recently utilized an optimized semi-localization by an adiabatic selective refocusing (semi-LASER) sequence^84,87^ and a study at 9.4T^91^ showed that 2HG can be reliably detected in patients *in vivo* using a short echo stimulated echo acquisition mode (STEAM) sequence, but such higher field strengths are not available in most hospitals potentially limiting the clinical utility of such sequences. Importantly however, these multiple approaches confirm the ability of MRS to clinically detect 2HG in brain tumor patients.

Further studies are needed in patient derived models. Nonetheless, given the feasibility of detecting 2HG in the clinic, and considering our findings that a drop in 2HG is associated with response to PI3K inhibition, this metabolite could serve as an indicator of drug target modulation in IDHmut glioma, providing an MRS detectable noninvasive imaging biomarker of response to PI3K/mTOR inhibitors that is independent of tumor size.

## Methods

### Cell culture and treatment

Two previously described genetically engineered IDHmut lines were used in this study: U87IDHmut and NHAIDHmut^24,92–95^. Briefly cells were created by introduction of lentiviral constructs encoding for R132H IDH1 cDNA into U87 GBM cells or immortalized normal human astrocytes (NHA) respectively^49^. Cells were routinely cultured as monolayers in DMEM (Gibco, ThermoFisher Scientific) supplemented with 10% FBS (Gibco, ThermoFisher Scientific), 100 units/mL penicillin and 100 μg/mL streptomycine (Gibco, ThermoFisher Scientific). Both cell lines were authenticated by short tandem repeat profiling within 6 months of any study. NHAIDHmut cells were treated with 32 μM XL765 (Voxtalisib, SAR245409; Selleck, USA) for 72 h and U87IDHmut cells were treated with 12 μM for 24 h. These time points and concentrations were chosen based on the doubling times of the NHAIDHmut (~40 h) and U87IDHmut (~20 h) such that cells were treated for approximately 1.5x their doubling time in order to achieve ~50% inhibition of cell proliferation. DMSO was used as vehicle control (final concentrations of 0.16% for NHAIDHmut cells and 0.06% for U87IDHmut). For ^13^C-MR studies, cells were cultured in medium (UCSF Cell Culture Facility) containing 5.5 mM [1-^13^C]glucose (Sigma-Aldrich) or 12.5 mM [2-^13^C]glucose (Cambridge Isotopes) out of 25 mM total glucose concentration, or 3 mM [3-^13^C]glutamine (Sigma-Aldrich) out of 6 mM total glutamine concentration.

### Western blot analysis

Cells and tumor tissues were examined as described previously^94,95^. Phosphorylated initiation factor 4E-binding protein 1 (p4E-BP1) and phosphorylated S6 kinase (pS6K) were used as a downstream readout of PI3K/mTOR signaling and β-actin as loading control. Control and treated cells and tissues were lysed using Cell Lysis Buffer (ThermoFisher Scientific) supplemented with 1 μl/ml protease inhibitor cocktail set III (Calbiochem) and 10 μl/ml phosphatases inhibitor (Sodium Orthovanadate; New England Biolabs, Inc.). Lysates normalized to cell number or to wet tissue weight were then run on 4–20% gels (Bio-Rad) using the SDS-PAGE method and electrotransferred onto nitrocellulose membranes. Membranes were blocked in blocking buffer containing 5% BSA in Tris-Buffered Saline Tween-20 (TBST) and incubated with the primary antibodies anti-p4E-BP1 (Thr37/46, Cell Signaling #2855), anti-4E-BP1 (Cell Signaling #9452), anti-pS6 kinase (Thr389, Cell Signaling #9234), anti-S6 kinase (Cell Signaling #9202) and anti-β-actin (Cell Signaling #4970) overnight at 4 °C. HRP-conjugated secondary antibodies (Cell Signaling #7074) were incubated for 60 min in TBST at room temperature. Immunocomplexes were visualized using ProSignal Pico (Genesee Scientific). Densitometry of the bands was performed using ImageJ software (NIH) to quantify protein expression levels and the data were normalized to the β-actin protein levels. Results are expressed as mean ± standard deviation (n ≥ 3 unless otherwise specified) and the significance of comparisons was determined using unpaired two-tailed Student’s t-test with unequal variance, with a p-value ≤ 0.05 considered as statistically significant.

### Cell extraction

Extractions were performed using the dual-phase extraction method^24^. Briefly, cells were trypsinized, washed with ice cold 0.85% saline (UCSF Cell Culture Facility) and 10 ml ice cold methanol (Sigma-Aldrich) was added to the cell pellet. The solution was then vortexed and 10 ml of ice-cold chloroform (Acros Organics) added. After another vortexing, 10 ml of ice cold Milli-Q water was added and a final vortexing performed. Phase separation was achieved by centrifugation for 10 min at 3000 rpm at 4 °C, phases separated and solvents removed by lyophylization. The aqueous phase was then reconstituted in 400 μl deuterium oxide (Acros Organics) for MRS studies.

### MRS data acquisition

^1^H and proton-decoupled ^13^C spectra of the aqueous phase of cell extracts were recorded using a 500 MHz spectrometer (Bruker BioSpin) equipped with a triple resonance cryoprobe. The ^1^H spectra were acquired using a 90° flip angle and 3 s repetition time (TR) while the ^13^C spectra were acquired using a 30° flip angle and TR of 3 s. For both control and XL765-treated extract samples there were 384 scans for the acquisition of the ^1^H spectra. In the case of ^13^C spectra, 4000 scans were acquired for controls and 12000 scans for XL765-treated extract samples (due to lower metabolite levels following treatment). In addition, fully relaxed ^1^H and ^13^C spectra were recorded and served to determine correction factors for saturation and nuclear Overhauser enhancement (NOE; ^13^C acquisitions only).

### MRS data analysis

#### Multivariate analysis

^1^H spectra were manually phased, baseline corrected and shifted to align TSP to 0 ppm in MestReNova (version 7.1.1, MestreLab Research). Peaks not associated with the metabolic profile (e.g. residual water (4.5–5.1 ppm), TSP and residual methanol were removed). Icoshift algorithm^96^ using Matlab (R2015b, The Mathworks Inc.) was applied to minimize peak misalignments and spectra were normalized to cell number and TSP area. An unsupervised Principle Component Analysis (PCA) was then performed using SIMCA (Version 15.0, Umetrics, Sweden) to determine the intrinsic clustering and distribution of samples between XL765-treated and control cells. In order to give equal weighting to all spectral regions, unit variance scaling was used. The orthogonal partial least squares discriminant analysis (OPLS-DA) supervised classification model was then applied to visualize class separation and the significant changes between groups were identified based on the S-plots. The validation of the PCA and OPLS-DA models against overfitting was determine by the goodness of fit (R2X: fraction of variation of X variables explained by PCA model and R2Y: fraction of variation of Y variables explained by OPLS-DA model) and goodness of prediction (Q2).

#### Univariate analysis

The concentration of metabolites identified via the multivariate analysis as most significantly altered following treatment (based on |r| > 0.8 in the S-plot) were also quantified using a targeted univariate analysis. Peaks were integrated using MestReNova, integrals corrected for saturation and NOE, and normalized to cell number and to an external sodium 3-(trimethylsilyl)propionate-2,2,3,3-d4 (TSP; Sigma-Aldrich) reference of known concentration. The significance of univariate comparisons was determined using an unpaired two-tailed Student’s t-test with unequal variance, with a p-value ≤ 0.05 considered as statistically significant.

### *In vivo* studies

All studies were performed in accordance with the National Institutes of Health Guide for the Care and Use of Laboratory Animals and were approved by the University of California San Francisco Institutional Animal Care and Use Committee (IACUC Protocol No: AN148391). Six-to-seven-week old female athymic nu/nu mice (Charles River) were intracranially injected with approximately 3 × 10^5^ U87IDHmut cells as previously^95^. Once tumors reached 2–3 mm in diameter (day zero, D0) the mice were randomized into either a treatment or a control group that were treated, respectively, with 30 mg/kg XL765 twice daily p.o. or with 10 mM HCl (vehicle facilitating drug uptake from the GI track). The XL765 solution was prepared immediately before administration.

### Anatomic MR imaging

All *in vivo* MR studies were performed using a 14.1 T vertical MR system (Agilent Technologies) equipped with a single channel volume ^1^H coil. A spin echo multi-slice sequence was used to acquire axial T2-weighted images of the mouse head in order to evaluate tumor volume. The sequence parameters were: echo time (TE) = 20 ms, TR = 1200 ms, field of view = 30 × 30 mm^2^, matrix = 256 × 256, slice thickness = 1 mm and number of averages = 2. Anatomical images were acquired on D0 and then every 3–4 days until animals needed to be sacrificed. Tumor volume was evaluated as the sum of manually contoured tumor areas in each slice multiplied by slice thickness using an in-house IDL based software^67^ and normalized to D0 value.

### *In Vivo*^1^H MRS

At the last time point prior to animal sacrifice ^1^H spectra were acquired from an 8 mm^3^ voxel placed in the center of the tumor using a point resolved spectroscopy (PRESS) sequence with: TE = 20 ms, TR = 4000 ms, NA = 448, 10000 points and spectral width 10000 Hz combined with water suppression. Additionally, a spectrum of the unsuppressed water signal was acquired with the same parameters as above, but with 4 averages only, for subsequent eddy current correction during LCModel spectral analysis. The spectral region between 0.5 and 4.0 ppm was then analyzed using LCModel^88^ and a basis set comprised of alanine (Ala), aspartate (Asp), total choline (tCho), total creatine (tCr), GABA, glutamine (Gln), glutamate (Glu), 2-hydroxyglutarate (2HG), myo-inositol (m-Ins), lactate (Lac), N-acetylaspartate (NAA), N-acetylaspartylglutamate (NAAG), phosphoethanolamine (PE), scyllo-inositol (s-Ins) and taurine (Tau). Each peak was normalized to the total signal calculated as the sum of all the quantified metabolites. The average concentration for each metabolite $\bar{C}$ was then calculated as follows (per the LCModel manual) so as to give values with lower Cramer-Rao lower bounds (CRLB) a higher weighting than values with a higher CRLB:

$$\bar{C}=\sum {w}_{j}{C}_{j}/\sum {w}_{j},$$

whereby: $${w}_{j}=1/{\sigma }_{j}^{2},\,{\sigma }_{j}={( \% CRLB)}_{j}\times {C}_{j}/100$$

with an estimated standard deviation

$$\sigma (\bar{C})=1/\sqrt{\sum {w}_{j}}$$

where ${C}_{j}$ the normalized signal of the metabolite for the animal *j* and ${( \% CRLB)}_{j}$ its corresponding CRLB expressed in per cent. Average metabolic changes were then calculated for each metabolite of interest using the lowest possible CRLB that led to at least 4 animals per group.

### Tumor tissue immunohistochemistry

At the end of the MR studies, tumor‐bearing brains were immediately removed and fixed in 10% buffered formalin for 24 h, then dehydrated with ethanol and embedded in wax (Paraplast Plus, McCornick Scientific). For each sample, adjacent tissue sections were stained with hematoxylin and eosin and Ki-67. For Ki-67, the slides were incubated for 60 min with a monoclonal mouse Ki‐67 antibody (M7240, Dako, Copenhagen, Denmark), diluted 1:100 at room temperature. Subsequently they were incubated for 60 min with the secondary antibody which was peroxidase‐labeled horseradish peroxidase polymer (Dako). The antigen localization was achieved by the 3,3’‐diaminobenzidine chromogen (Dako). Nuclei were considered to be Ki-67 positive if any nuclear staining was present, regardless of staining intensity. A Ki-67 labeling index was defined as the average percentage of positive stained cells from 3 regions of each sample.

### Analysis of tumor tissue

#### Extraction

At the end of the MR studies, tumors were excised, snap-frozen in liquid nitrogen and stored at −80 °C until further investigation. For complementary ^1^H MRS studies pieces of tumor tissue weighting 9 to 24 mg (wet mass) were homogenized in 500 μl ice cold phosphate buffer (PBS) with 1 μl/ml protease inhibitor cocktail set III (Calbiochem) using beads (TissueLyser LT, QIAGEN). The dual-phase extraction method described above for the cell extracts was then applied and the aqueous phase was reconstituted in 400 μl 100 mM phosphate buffer in deuterium oxide (Acros Organics).

#### MRS data acquisition and analysis

^1^H spectra of tumor tissue extracts were recorded and analyzed the same way as described above for the cell extracts. The only modification was that the analysis was focused in the *in vivo* relevant spectral region of 0.5 to 4.2 ppm.

## Supplementary information

Supplementary File

## Data Availability

Data generated during the current study are available from the corresponding author upon request.
