# Supplementary material for: PI3K/mTOR inhibition of IDH1 mutant glioma leads to reduced 2HG production that is associated with increased survival
Source: Sci Rep. 2019 Jul 19;9:10521. doi: 10.1038/s41598-019-47021-x (PMC6642106; doi:10.1038/s41598-019-47021-x)
Supplement: Supplementary file 1 — Supplementary File [file 41598_2019_47021_MOESM1_ESM.docx]

**PI3K/mTOR inhibition of IDH1 mutant glioma leads to reduced 2HG production that is associated with increased survival**

Georgios Batsios, Pavithra Viswanath, Elavarasan Subramani, Chloe Najac, Anne Marie Gillespie, Romelyn Delos Santos, Abigail R Molloy, Russell O Pieper, Sabrina M. Ronen*

------------------------------------

Georgios Batsios^a^, Pavithra Viswanath^a^, Elavarasan Subramani^a^, Chloe Najac^a^, Anne Marie Gillespie^a^, Romelyn Delos Santos^a^, Abigail R Molloy^a^, Russell O Pieper^b^, Sabrina M. Ronen🖂^a,c^

^a^Department of Radiology and Biomedical Imaging, Mission Bay Campus, 1700 4th Street, Byers Hall, University of California, 94158, San Francisco, CA, United States

^b^Department of Neurological Surgery, Helen Diller Research Center, 1450 3rd Street, University of California, 94143, San Francisco, CA, United States

^c^Brain Tumor Research Center, Helen Diller Family Cancer Research Building, 1450 3rd Street, University of California, 94158, San Francisco, CA, United States

*Correspondence and requests for materials should be addressed to S.M.R. (email: sabrina.ronen@ucsf.edu)

**
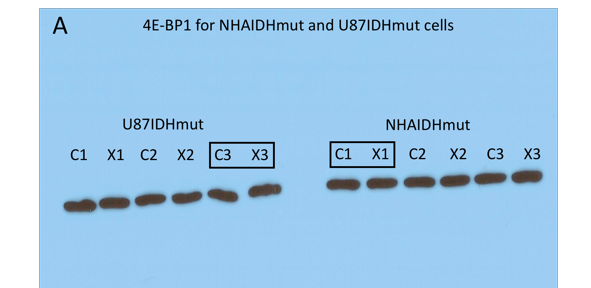
**

**
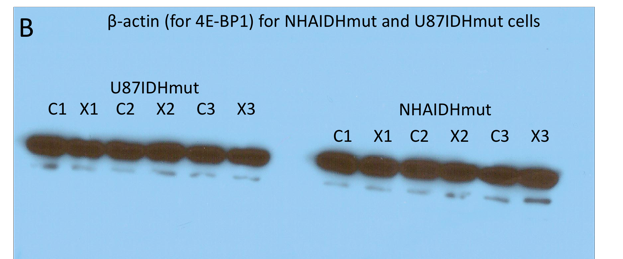
**

**
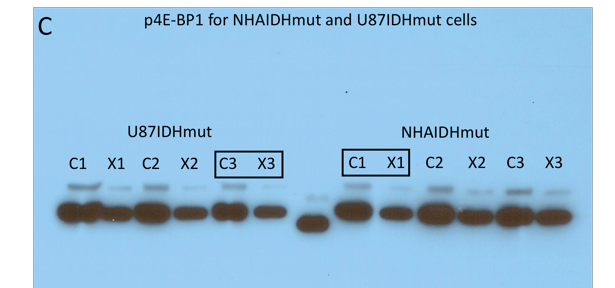
**

**
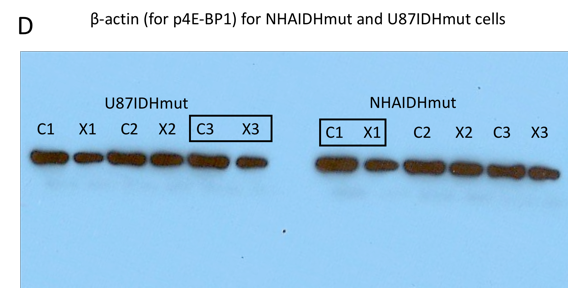
**

**Supplementary Figure 1.** Uncropped western blots of 4E-BP1 (**A**; 15kDa), β-actin for 4E-BP1 (**B**; 42kDa), phosphorylated 4E-BP1 (**C**; 15kDa) and β-actin for p4E-BP1 (**D**; 42kDa) for NHAIDHmut and U87IDHmut model for control (samples marked as C1, C2, C3) and XL765 (samples marked as X1, X2, X3) treatment. The black box marks the samples that are presented in Figure 1.

**
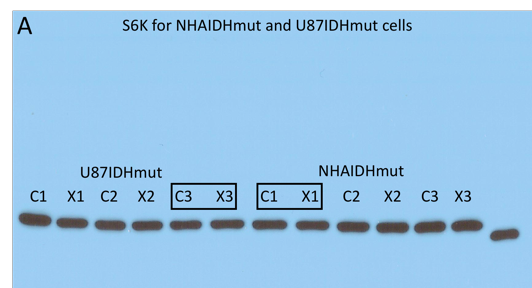
**

**
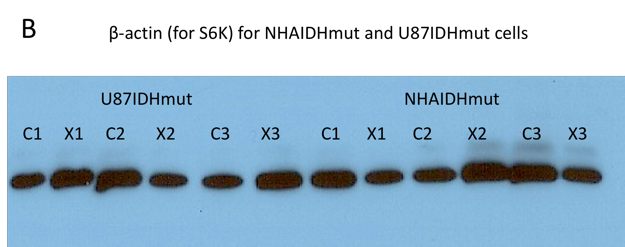
**

**
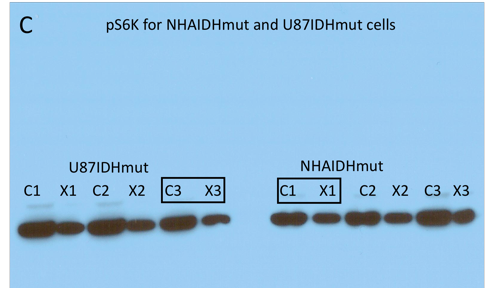
**

**
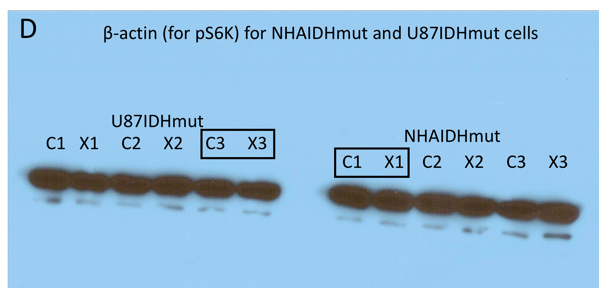
**

**Supplementary Figure 2.** Uncropped western blots of S6K (**A**; 70kDa), β-actin for S6K (**B**; 42kDa), phosphorylated S6K (**C**; 70kDa) and β-actin for pS6K (**D**; 42kDa) for NHAIDHmut and U87IDHmut model for control (samples marked as C1, C2, C3) and XL765 (samples marked as X1, X2, X3) treatment. The black box marks the samples that are presented in Figure 1.


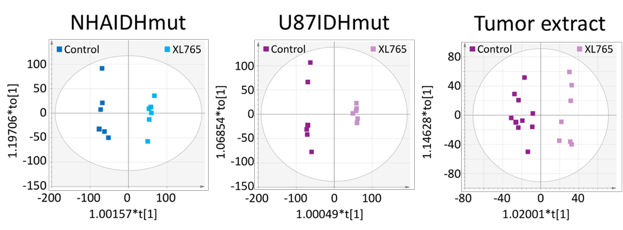


**Supplementary Figure 3.** Two-dimensional scatter plot of orthogonal partial least squares discriminant analysis (OPLS-DA) shows discrimination of control (dark blue: NHAIDHmut cells and dark purple: U87IDHmut cells and U87IDHmut tumor extracts) and XL765-treated (light blue: NHAIDHmut cells and light purple: U87IDHmut cells and U87IDHmut tumor extracts) groups.

**
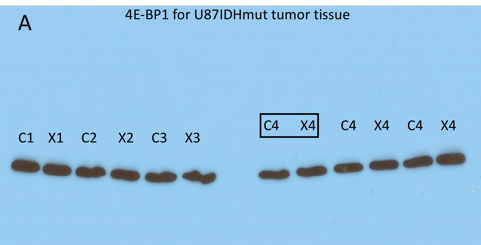
**

**
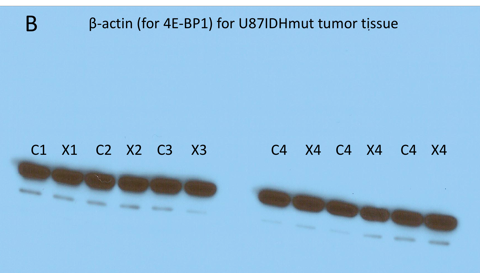
**

**
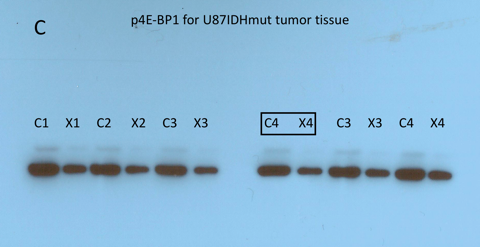
**

**
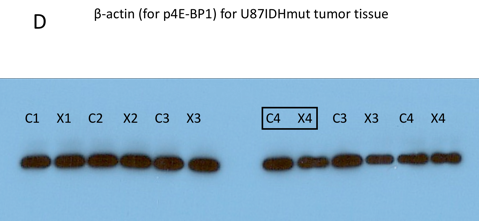
**

**Supplementary Figure 4.** Uncropped western blots of 4E-BP1 (**A**; 15kDa), β-actin for 4E-BP1 (**B**; 42kDa), phosphorylated 4E-BP1 (**C**; 15kDa) and β-actin for p4E-BP1(**D**; 42kDa) for U87IDHmut tumors for control (samples marked as C1, C2, C3, C4) and XL765 (samples marked as X1, X2, X3, X4) treatment. The black box marks the samples that are presented in Figure 4.


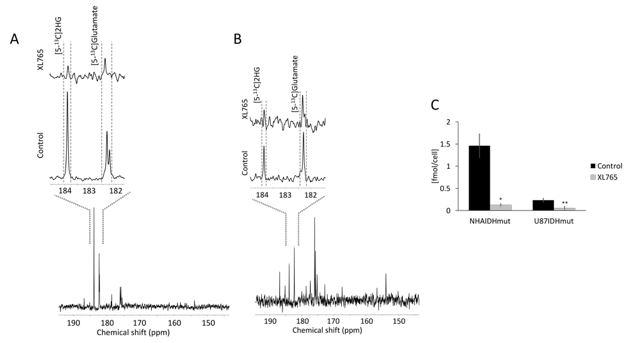


**Supplementary Figure 5.** Glucose flux to 2HG is reduced in NHAIDHmut and U87IDHmut cells under XL765 treatment. **(A & B)** Representative ^13^C-spectra ([2-^13^C]glucose labeling) of NHAIDHmut and U87IDHmut control cells respectively. Inserts: Expansion of [5-^13^C]2HG and [5-^13^C]glutamate region for XL765-treated (top) and control (bottom). **(C)** Average 2HG produced from [2-^13^C]glucose for control and XL765-treated NHAIDHmut cells and U87IDHmut. Black bar: Control; Grey bar: XL765-treated.
